# TLQP-21 is a low potency partial C3aR activator on human primary macrophages

**DOI:** 10.3389/fimmu.2023.1086673

**Published:** 2023-01-26

**Authors:** Xaria X. Li, John D. Lee, Han S. Lee, Richard J. Clark, Trent M. Woodruff

**Affiliations:** School of Biomedical Sciences, The University of Queensland, St. Lucia, Australia

**Keywords:** TLQP-21, Complement C3a, C3aR, C5aR1, C5aR2

## Abstract

TLQP-21 is a 21-amino acid neuropeptide derived from the VGF precursor protein. TLQP-21 is expressed in the nervous system and neuroendocrine glands, and demonstrates pleiotropic roles including regulating metabolism, nociception and microglial functions. Several possible receptors for TLQP-21 have been identified, with complement C3a receptor (C3aR) being the most commonly reported. However, few studies have characterised the activity of TLQP-21 in immune cells, which represent the major cell type expressing C3aR. In this study, we therefore aimed to define the activity of both human and mouse TLQP-21 on cell signalling in primary human and mouse macrophages. We first confirmed that TLQP-21 induced ERK signalling in CHO cells overexpressing human C3aR, and did not activate human C5aR1 or C5aR2. TLQP-21 mediated ERK signalling was also observed in primary human macrophages. However, the potency for human TLQP-21 was 135,000-fold lower relative to C3a, and only reached 45% at the highest dose tested (10 μM). Unlike in humans, mouse TLQP-21 potently triggered ERK signalling in murine macrophages, reaching near full activation, but at ~10-fold reduced potency compared to C3a. We further confirmed the C3aR dependency of the TLQP-21 activities. Our results reveal significant discrepancy in TLQP-21 C3aR activity between human and murine receptors, with mouse TLQP-21 being consistently more potent than the human counterpart in both systems. Considering the supraphysiological concentrations of hTLQP-21 needed to only partially activate macrophages, it is likely that the actions of TLQP-21, at least in these immune cells, may not be mediated by C3aR in humans.

## Introduction

VGF (non-acronym) is a large neuropeptide precursor (~68 kDa) classified as a member of the extended granin protein family (1). VGF is cleaved by the prohormone convertases such as prohormone convertase (PC) 1/3 and PC2 to generate a variety of bioactive peptides of low molecular weights (2). Among them, TLQP-21 is a 21-residue peptide derived from the *C*-terminus (residues 556-576) of the VGF precursor protein (3). Using the expression of VGF mRNA as a proxy, past studies have suggested the expression of TLQP-21 in the central and peripheral nervous system and in several neuroendocrine glands including the pancreas, adrenal medullar and pituitary, however, further investigation using more specific molecular tools are needed to confirm these findings (2–4). TLQP-21 possesses pleiotropic roles in physiology and cell biology, including reducing obesity by decreasing food intake and/or increasing energy expenditure; potentiating adrenergic-induced lipolysis in adipocytes; modulating gastric contractility and acid secretion; reproduction; nociception; neuroprotection and modulation of microglia functions (2, 3).

In recent years, three possible binding targets of TLQP-21 have been identified: the globular C1q receptor (5, 6), heat shock cognate 71 kDa protein A8 (HSPA8) and the complement C3a receptor (C3aR) (1, 7), with C3aR being the most extensively studied (2, 8). C3aR is a classical G protein-coupled receptor which is the cognate receptor for the complement peptide C3a (9). From a cell signalling perspective, TLQP-21 ligation to cells activates phospholipase C (PLC)-β mediated generation of diacylglycerol (DAG) and inositol 1,4,5-trisphosphate (IP_3_), which subsequently triggers extracellular signal-regulated kinase (ERK)1/2 phosphorylation and intracellular calcium mobilisation (10). These signalling pathways have been demonstrated in Chinese hamster ovary (CHO), 3T3-L1 and murine adipocytes, RAW264.7 macrophages and primary murine microglia to be dependent on C3aR (6, 11–14).

C3aR is expressed by all leukocytes of myeloid origin, such as neutrophils, dendritic cells, microglia and macrophages, and has been shown to modulate multiple aspects of immune cell signalling and functions in response to C3a (9, 15). However, despite this widespread expression of C3aR on myeloid cells, there is a general lack of studies examining the effect of TLQP-21 on these immune cells. Therefore, in the present study, we aimed to define the pharmacological activity of TLQP-21 on the cell signalling of human and murine primary macrophages. Human TLQP-21 (hTLQP-21) and mouse TLQP-21 (mTLQP-21) harbour 24% (5 out of 21) difference in their amino acid sequences (2, 7). Considering the differential activities of the two molecules as reported previously (16, 17), both hTLQP-21 and mTLQP-21 were synthesised, and comparatively analysed. We identified that both versions of TLQP-21 induced ERK signalling in primary human and murine macrophages at relatively high concentrations compared to C3a. Signalling by TLQP-21 was C3aR-dependent, with mouse TLQP-21 being consistently more potent in both mouse and human cells, relative to the human peptide. In view of the relatively low C3aR activity of human TLQP-21 we observed on macrophages, this action of TLQP-21 may not be physiologically relevant in human peripheral immune cells such as macrophages.

## Materials and methods

### Ligands and materials

Human C5a, human TLQP-21 (TLQPPSALRRRHYHHALPPSR) and mouse TLQP-21 (TLQPPASSRRRHFHHALPPAR) were synthesized by Fmoc-based solid phase peptide synthesis using previously described methods (18, 19). The C3aR inhibitor, SB290157 trifluoroacetate salt, and purified human C3a were purchased from Merck (Perth, Australia). Recombinant mouse C3a was purchased from R&D Systems (Minneapolis, USA). Bovine serum albumin (BSA) was purchased from Merck (Perth, Australia). For cell culture, trypsin-EDTA, HBSS, HEPES, Dulbecco’s Modified Eagle’s Medium (DMEM), phenol-red free DMEM, Ham’s F12, Iscove’s Modified Dulbecco’s Medium, RPMI-1640 and Penicillin-Streptomycin were purchased from Thermo Fisher Scientific (Melbourne, Australia). Dulbecco’s phosphate-buffered saline was purchased from Lonza (Melbourne, Australia). Stocks of TLQP-21 were reconstituted in water at 1 mM. All ligand dilutions were prepared in serum-free medium (SFM) containing 0.1% BSA.

### Cell culture

Cell lines for this study were cultured as previously described (20). Non-transfected CHO-K1 cells or CHO cells stably expressing the human C3aR (CHO-C3aR; Product # ES-730-C, PerkinElmer, Melbourne, Australia) were maintained in Ham’s F12 medium containing 10% foetal bovine serum (FBS), 100 IU/ml penicillin and 100 μg/ml streptomycin, with additional 400 μg/ml G418 (*In vivo*Gen, San Diego, USA) added for CHO-C3aR cell culture. HEK293 cells were maintained in DMEM medium containing 10% FBS, 100 IU/ml penicillin and 100 μg/ml streptomycin. All cell lines were maintained in T175 flasks (37°C, 5% CO_2_) and subcultured at 80-90% confluency using 0.05% trypsin-EDTA in DPBS.

HMDMs were generated and cultured as previously described (20), with experiments approved by The University of Queensland Human Research Ethics Committee. Briefly, human buffy coat blood from anonymous healthy donors was obtained through the Australian Red Cross Blood Service (Brisbane, Australia). Human CD14+ monocytes were isolated from blood using Lymphoprep density centrifugation (STEMCELL, Melbourne, Australia) followed by CD14+ MACS magnetic bead separation (Miltenyi Biotec, Sydney, Australia). The isolated monocytes were differentiated for 7 days in Iscove’s Modified Dulbecco’s Medium supplemented with 10% FBS, 100 IU/ml penicillin, 100 μg/ml streptomycin and 15 ng/ml recombinant human macrophage colony stimulating factor (BioLegend, San Diego, USA) on 10 mm square dishes (Bio-strategy, Brisbane, Australia). Non-adherent cells were removed by washing with DPBS, and the adherent differentiated HMDMs were harvested by gentle scraping.

Mouse bone marrow-derived macrophages (BMDMs) were obtained and cultured as previously described (20, 21). All experiments using mice were approved by the University of Queensland animal ethics committee. Wild-type and C3aR^-/-^ mice on a C57BL/6J genetic background (*n* = 3) were sacrificed by cervical dislocation. The tibia was removed and sterilised. Upon removal of both epiphyses, bone marrow cells were harvested by flushing the central cavity with complete RPMI-1640 medium using a 10 ml syringe attached to a 25-gauge needle. Cells were then cultured in complete RPMI-1640 medium (containing 10 % FBS, 100 IU/ml penicillin, 100 μg/ml streptomycin) supplemented with 100 ng/ml recombinant human macrophage colony stimulating factor on 10 mm square dishes. Mature adherent macrophages for assays were harvested on day 6-7 by gentle scraping.

### Phospho-ERK1/2 assays

Ligand-induced ERK1/2 phosphorylation was assessed using the AlphaLISA *Surefire Ultra* p-ERK1/2 (Thr202/Tyr204) kit (PerkinElmer, Melbourne, Australia) following the manufacturer’s protocol as previously described (18, 20, 22). Briefly, CHO-K1, CHO-C3aR, CHO-C5aR1, HMDMs (50,000/well) or BMDMs (90,000/well) were seeded in tissue culture-treated 96-well plates (Corning, Corning, USA) for 24 h and serum-starved overnight. All ligand dilutions were prepared in serum-free medium (SFM) containing 0.1% BSA. For inhibition assays, cells were pre-treated with the following ligands (5 µM SB290157, or respective concentrations of hC3a, mC3a, hC5a, hTLQP-21, and mTLQP-21) or the solvent-only control for 30 min before agonist (hC3a, hTLQP-21 and mTLQP-21) addition. The concentration and identity of the ligands are described in detail in the figure legends. For stimulation, cells were treated with ligands at the indicated concentrations for 10 min at RT and then immediately lysed using AlphaLISA lysis buffer on a microplate shaker (450 rpm, 10 min). For the detection of phospho-ERK1/2 content, cell lysate (5 μL/well) was transferred to a 384-well ProxiPlate (PerkinElmer, Melbourne, Australia) and added to the donor and acceptor reaction mix (2.5 μL/well, respectively), followed by a 2-h incubation at RT in the dark. On a Tecan Spark 20M (Tecan, Männedorf, Switzerland), the plate was measured using standard AlphaLISA settings.

### BRET assays measuring β-arrestin 2 recruitment to C5aR2

The C5a-mediated β-arrestin 2 recruitment to C5aR2 was measured using bioluminescence resonance energy transfer (BRET)-based assay using methods described elsewhere (20, 22, 23). Briefly, HEK293 cells were transiently transfected with β-arrestin 2-NanoLuc (Nluc) and human C5aR2-Venus constructs using XTG9 (Roche, Sydney, Australia) for 24 h. Transfected cells were then seeded (100,000/well) onto white 96-well plates (Corning, New York, USA) in phenol-red free DMEM containing 5% FBS overnight. For BRET assay, cells were firstly incubated with the substrate Endurazine (1:200, Promega, Sydney, Australia) for 2 h (37°C, 5% CO_2_). All ligands were prepared in SFM containing 0.1% BSA. On a Tecan Spark 20M microplate reader (Tecan, Männedorf, Switzerland) (37°C), the BRET light emissions (460-485 and 520-545 nm) were continuously monitored for 24 reads with respective ligands (C5a, hTLQP-21, m-TLQP-21) and vehicle control (medium) added after the first 4 reads. The ligand-induced BRET ratio was calculated by subtracting the emission ratio of Venus (520-545 nm)/Nluc (460-485 nm) of the vehicle-treated wells from that of the ligand-treated wells, and the data at 40 min post ligand addition was used for the plotting of concentration-response curves.

### Data collection, processing and analysis

All experiments were conducted in triplicates and repeated on three separate occasions unless otherwise specified. Data was analysed using GraphPad software (Prism 9.1). Data from each individual repeat was normalised accordingly (as specified in the figure legends) before being combined and expressed as mean ± standard error of the mean (S.E.M.) unless otherwise described. For all dose-response studies, logarithmic concentration-response curves were plotted using combined data and analysed to determine the respective potency values. Statistical analysis was performed using two-way ANOVA with Dunnett’s *post hoc* analysis. Donor-matched comparisons were made for HMDM experiments. Differences were deemed significant when *p* < 0.05.

## Results

### hTLQP-21 and mTLQP-21 activate ERK signalling in CHO-K1 and CHO-C3aR cells

**Figure 1:**
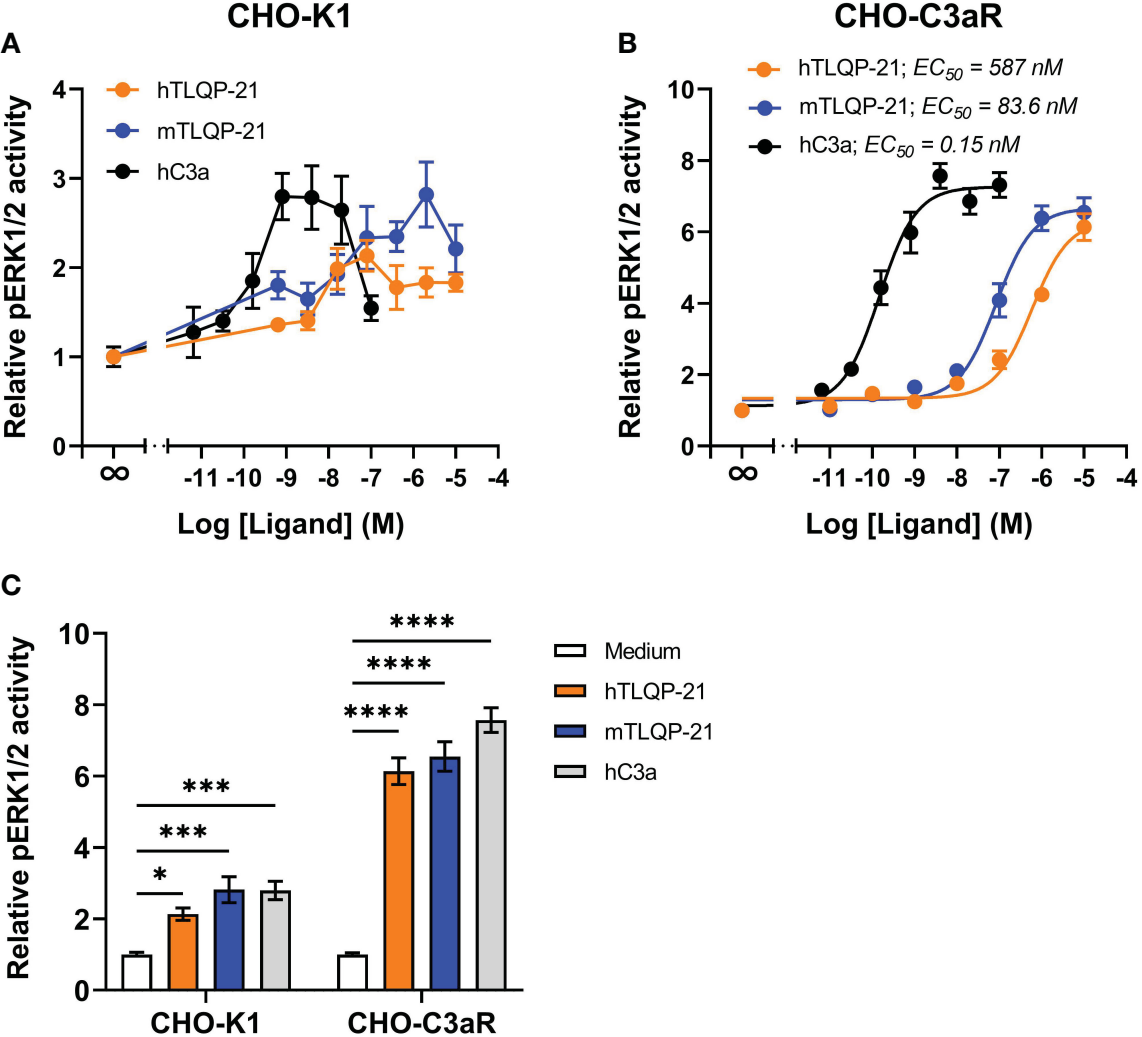
hTLQP-2 and mTLQP-21 activate ERK signalling in CHO-K1 and CHO-C3aR cells. hTLQP-21, mTLQP-21 and plasma-derived human C3a were tested in **(A)** non-transfected CHO-K1 or **(B)** CHO cells stably expressing human C3aR. CHO cells were serum-starved overnight and then stimulated with various ligands for 10 min before being lysed. The phospho-ERK1/2 content in the lysate was measured and expressed as fold-baseline before being combined. The maximum relative pERK1/2 activity induced by each ligand is shown in **(C)**. Data represent mean ± S.E.M. of triplicate measurements from 3-4 independent experiments (n = 3-4). Two-way ANOVA with Dunnett’s *post hoc* analysis. *P < 0.05, ***P < 0.001, ****P < 0.0001. Ligand treated versus medium treated cells for each cell line.

We first tested hTLQP-21 and mTLQP-21 in CHO-K1 cells to confirm the prior reported agonistic activities of these peptides in this cell line. Receptor-induced ERK1/2 phosphorylation was used as a readout. Plasma-derived human C3a (hC3a) significantly triggered ERK1/2 phosphorylation in CHO-K1 cells (**Figures 1A, C**), confirming the expression of endogenous C3aR in these cells (7). A significant reduction in ERK1/2 phosphorylation was observed at higher C3a concentrations, similar to the previous reported data for primary human macrophages (18). Both human and mouse versions of TLQP-21 triggered ERK signalling in CHO-K1 cells, albeit with lower efficacy and potency relative to hC3a. We next examined the ERK-inducing activities of TLQP-21 in CHO cells overexpressing human C3aR (**Figures 1B, C**; **Table 1**). hC3a potently and dose-dependently triggered ERK1/2 phosphorylation in CHO-C3aR cells (EC_50_ = 0.15 nM). Both hTLQP-21 and mTLQP-21 acted as full agonists relative to C3a on this signalling pathway, with mTLQP-21 being ~ 7-fold more potent than hTLQP-21 (EC_50_ = 83.6 nM for mTLQP-21 and 587 nM for hTLQP-21, respectively). Notably, the relative efficacy of C3a-induced ERK signalling in CHO-K1 cells was 2.6-fold lower than that in CHO-C3aR cells (2.7- versus 7.3-fold baseline for CHO-K1 and CHO-C3aR, respectively; **Figure 1C**). This is likely attributed to the much lower endogenous C3aR expression in the non-transfected CHO cells (7, 20).

**Table 1:** Summary of potencies and activities of TLQP-21 tested on CHO-C3aR, HMDM and BMDM.

	CHO-C3aR			HMDM			BMDM		
	EC_50_ (nM)	logEC_50_ ± SE	% C3a activity ± SE	EC_50_ (nM)	logEC_50_ ± SE	% C3a activity ± SE	EC_50_ (nM)	logEC_50_ ± SE	% C3a activity ± SE
**C3a**	0.15	-9.8 ± 0.1	100 ± 2.8	0.11	-9.9 ± 0.1	100 ± 6.2	8.4	-8.1 ± 0.1	100 ± 4.1
**hTLQP-21**	587.0	-6.2 ± 0.1	87.0 ± 3.5	14820	-4.8 ± 0.9	45.3 ± 10	401	-6.4 ± 0.2	63.3 ± 4.2
**mTLQP-21**	83.6	-7.1 ± 0.1	91.8 ± 2.7	2846	-5.5 ± 0.2	93.5 ± 18	96.5	-7.0 ± 0.3	80.1 ± 5.1

Potencies and efficacies of human and mouse TLQP-21 were determined in CHO-C3aR, HMDM and BMDM cells using phospho-ERK1/2 assays. Data were normalised to fold baseline before combined for analysis (n = 3-4).

### TLQP-21 does not activate human C5aR1 or C5aR2

**Figure 2:**
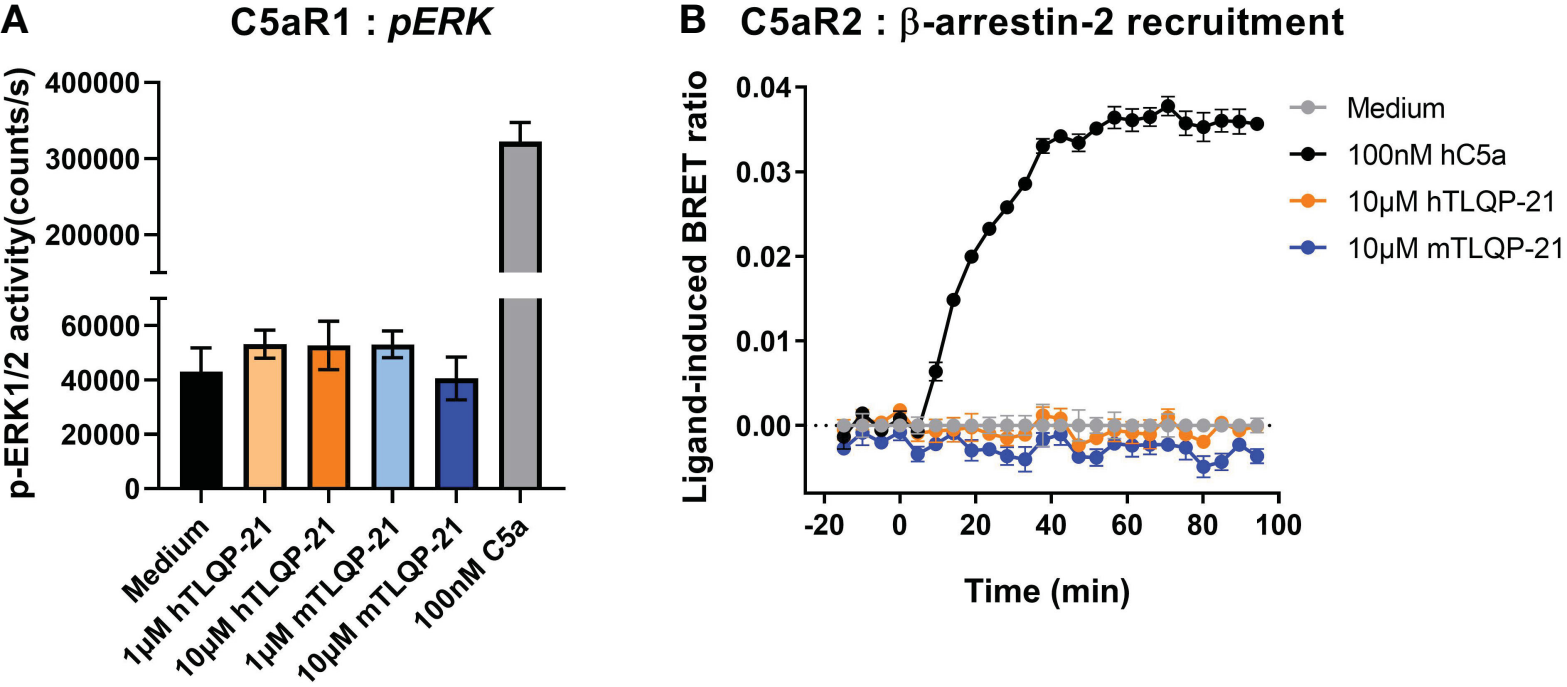
hTLQP-2 and mTLQP-21 do not activate human C5aR1 or C5aR2. **(A)** CHO cells stably expressing human C5aR1 were serum-starved overnight and then stimulated with various ligands for 10 min before being lysed. The phospho-ERK1/2 content in the lysate was measured and expressed as counts/s. **(B)** HEK293 cells were transiently transfected using C5aR2-Venus and β-arrestin 2-nanoluc BRET pairs for 24 hours, and seeded overnight. Filtered light emissions between 460-485 nm (nanoluc) and 520-545 nm (Venus) were continually monitored for 90 min, with respective ligands (mTLQP-21 and hTLQP-21, 10 μM; hC5a, 100 nM) added at the 0 min time point. Data are expressed as ligand-induced BRET (Venus/nanoLuc emission) ratios. Data represent mean ± S.E.M. of triplicate measurements from a single experiment.

We have previously reported the promiscuous activity of complement receptor peptides across the three closely related anaphylatoxin receptors C3aR, C5aR1 and C5aR2 (9, 20). We therefore next examined if TLQP-21 may also demonstrate bioactivity at C5a receptors. C5aR1 is a classical GPCR, which, upon activation, recruits Gα_i_ and Gα_16_, and triggers ERK1/2 phosphorylation (9, 22). However, C5aR2, being a non-canonical GPCR, does not couple to the common classes of G proteins and is devoid of the classical G protein-mediated signalling activities (24–26). We therefore measured ligand-induced β-arrestin 2 recruitment as a readout for C5aR2 activation, utilising a BRET assay previously established in HEK293 cells (23, 27). Both human and mouse TLQP-21, up to 10 μM, did not trigger significant ERK1/2 activation in CHO cells overexpressing human C5aR1 (**Figure 2A**), nor could we detect any induced C5aR2-mediated β-arrestin 2 recruitment in the BRET assay (**Figure 2B**). Therefore, among the three complement receptors examined, the activity of TLQP-21 on C3aR is likely to be selective.

### TLQP-21 triggers C3aR-dependent ERK signalling in human monocyte-derived macrophages

**Figure 3:**
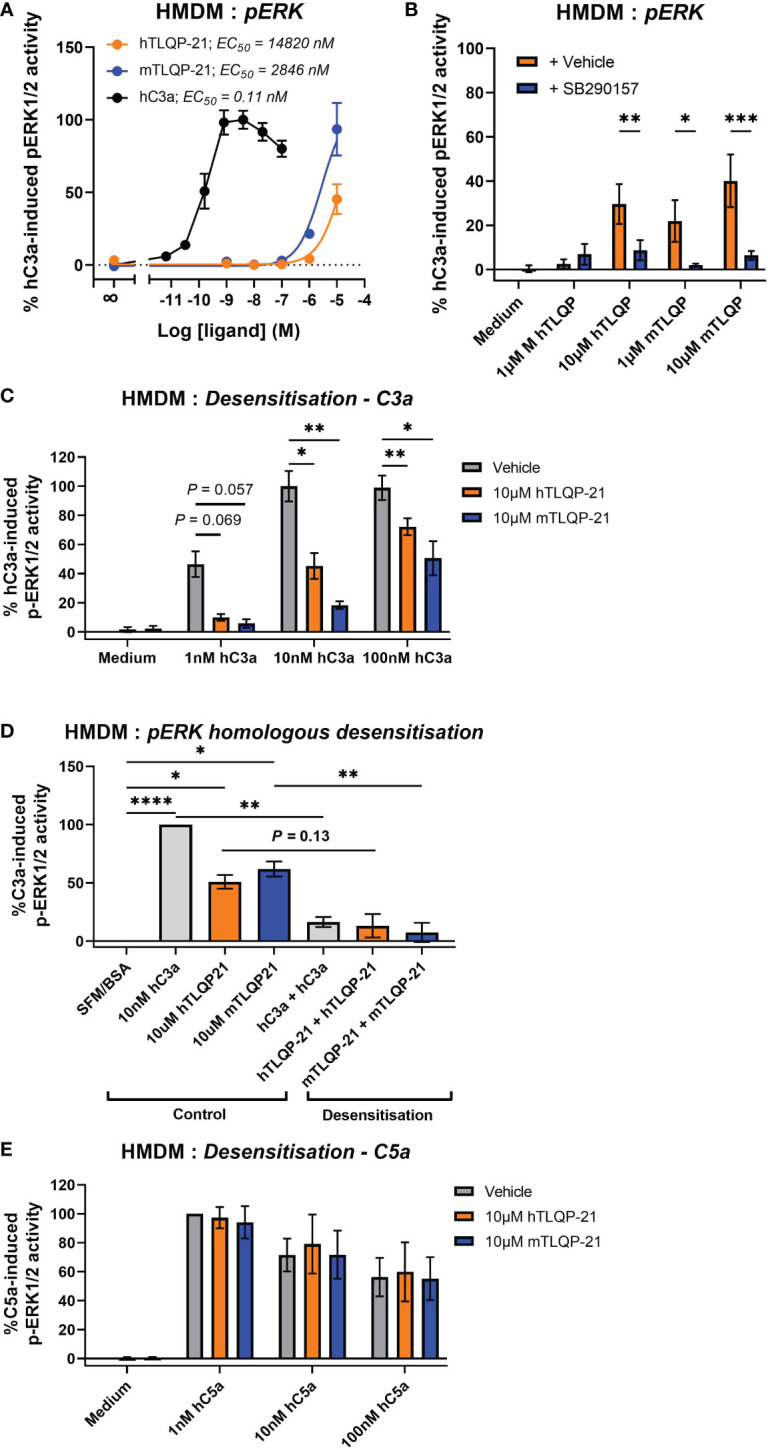
Agonistic and antagonistic activities of hTLQP-21 and mTLQP-21 in human monocyte-derived macrophages. Serum-starved HMDMs (50,000/well) were **(A)** stimulated with medium or respective ligands at the indicated concentrations for 10 min and then lysed, or **(B)** pre-treated with 5 µM SB290157 for 30 min prior to stimulation. **(C)** HMDMs were pre-treated with 10 μM hTLQP-21 or mTLQP-21 for 30 min before being stimulated with respective concentrations of human C3a. **(D)** HMDMs were pre-treated with 10 nM hC3a, 10 μM hTLQP-21 or mTLQP-21 for 30 min before being stimulated with the same concentrations of the above ligands for 10 min and then lysed. **(E)** HMDMs were pre-treated with 10 μM hTLQP-21 or mTLQP-21 for 30 min before being stimulated with respective concentrations of human C5a for 10 min and then lysed. The phospho-ERK1/2 content in the cell lysate was measured and normalised to the maximum hC3a/hC5a-induced levels before being combined. Data represent mean ± S.E.M. of triplicate measurements using cells from 4 independent donors (n = 4). Two-way ANOVA with Dunnett’s *post hoc* analysis. *P < 0.05, **P < 0.01, ***P < 0.001, ****P < 0.0001. Ligand pre-treated *versus* vehicle treated donor-matched cells.

Next, we aimed to determine the effect of TLQP-21 treatment in primary human monocyte-derived macrophages (HMDMs), which is an established and widely used model of resting tissue macrophages that expresses high levels of endogenous human C3aR (20, 28, 29). We stimulated the cells with different concentrations of hTLQP-21 and mTLQP-21 and then detected the phosphorylated ERK1/2 content in the cell lysate. Expectedly, human C3a potently induced ERK1/2 phosphorylation in HMDMs with an EC_50_ of 0.11 nM (**Figure 3A**; **Table 1**). Both versions of TLQP-21 triggered ERK signalling in HMDMs, but with greatly reduced potencies (by 25,000- to 135,000-fold) in comparison to hC3a (estimated EC_50_ = 2.8 μM for mTLQP-21 and 14.8 μM for hTLQP-21 respectively). In addition, whilst mTLQP-21 at 10 μM triggered up to 93% of the hC3a-induced level, the ERK signalling activity induced by hTLQP-21 only reached 45%. In order to examine whether this low-potency TLQP-21 induced ERK activation was dependent on C3aR, we pre-treated HMDMs with the C3aR antagonist SB290157 (30). A 5 μM concentration of SB290157 was chosen to avoid any C3aR or C5aR2 agonistic effect (20). SB290157 significantly inhibited hTLQP-21 and mTLQP-21 induced ERK signalling in HMDMs by 70-90 % (**Figure 3B**). The antagonising activity of SB290157 was more potent towards mTLQP-21 induced ERK activation (to 16% of the control level) than that of hTLQP-21 (to 29% of the control level), and dependent on the concentration of TLQP-21 tested. Notably, under control conditions, 10 μM mTLQP-21 only reached 40% of the C3a induced activity (**Figure 3B**) rather than the 93% as identified in **Figure 3A**. This discrepancy is likely caused by the different C3aR expression levels between individual human donors (29), which could affect the relative activities of the ligands amongst separate experiments.

To further validate the C3aR dependency of TLQP-21 in human macrophages, we conducted a desensitization assay using hC3a, based on the concept that any TLQP-21 binding to C3aR would impede hC3a from binding and activating C3aR, and the ERK1/2 phosphorylation triggered by TLQP-21 would desensitise subsequent ERK signalling triggered by hC3a. We observed that pre-treating cells with both versions of TLQP-21 significantly dampened hC3a-induced ERK signalling, with mTLQP-21 being consistently more potent than hTLQP-21 (**Figure 3C**). This inhibitory effect is likely to be competitive as it could be overcome by increasing the concentration of hC3a (ranging from 80-90% inhibition for 1 nM hC3a and 30-50% for 100 nM hC3a). In homologous desensitisation control experiments (**Figure 3D**), as expected, pretreating cells with hC3a, hTLQP-21 and mTLQP-21 significantly dampened the subsequent ERK activity triggered by these ligands. No similar TLQP-21 mediated inhibition was observed for hC5a-indued ERK1/2 activity (**Figure 3E**), indicating the inhibitory activity of TLQP-21 was C3aR-specific rather than towards basal activities of the cell. Based on these data, we conclude that both human and mouse TLQP-21 induce C3aR-mediated ERK signalling in HMDMs.

### TLQP-21 triggers C3aR-dependent ERK signalling in murine bone marrow-derived macrophages

**Figure 4:**
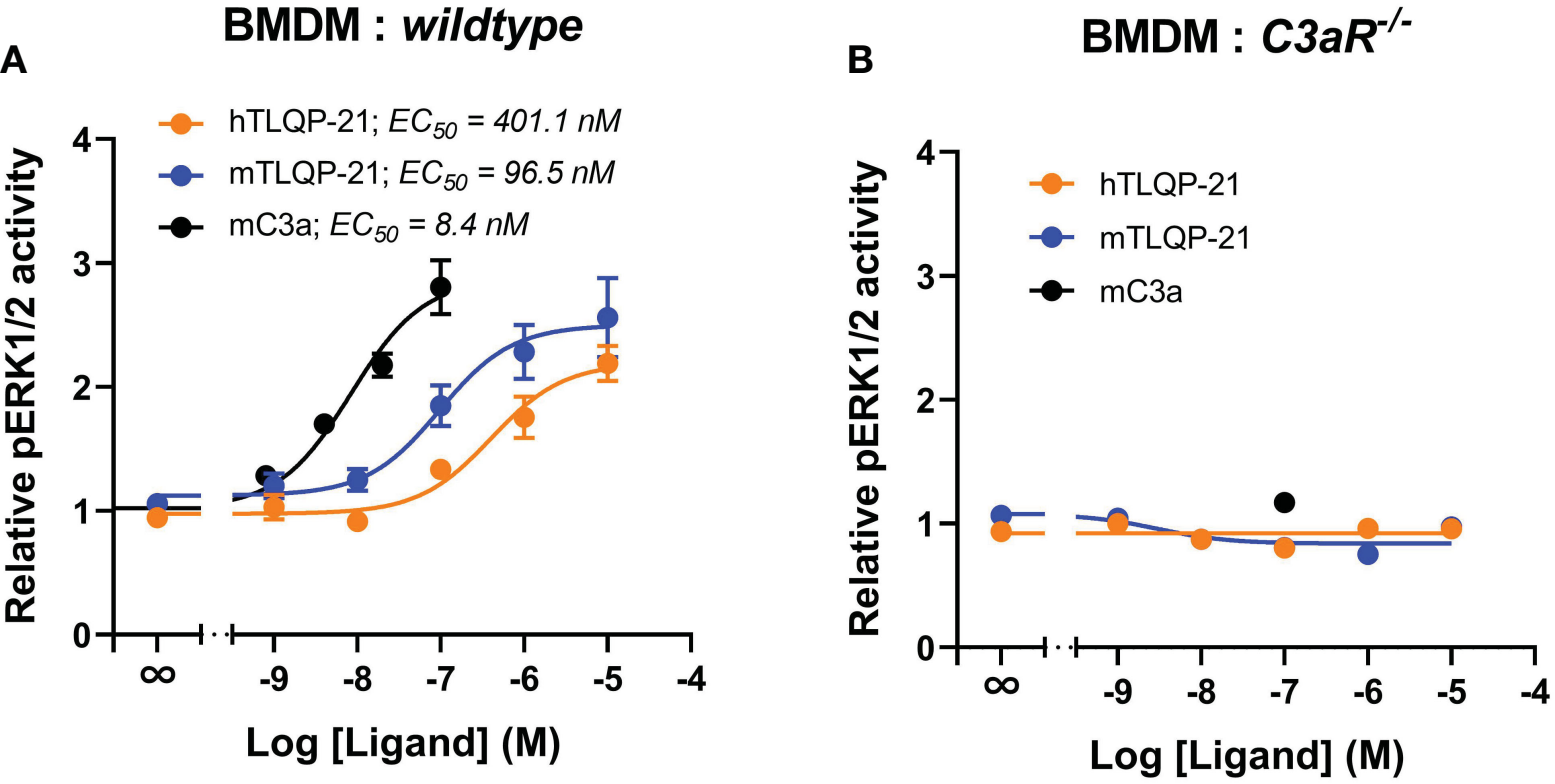
TLQP-21 triggers ERK signalling through C3aR in murine bone marrow-derive macrophages. BMDMs (90,000/well) from *wildtype mice* **(A)** or with *C3aR knockout mice* **(B)**, were serum-starved overnight and then stimulated with respective ligands at the indicated concentrations for 10 min. The phospho-ERK1/2 content in the cell lysate was measured and normalised to the medium only-treated levels before being combined. Data represent mean ± S.E.M. of triplicate measurements using cells from 3 mice (n = 3).

We next assessed if TLQP-21 similarly activated ERK signalling in murine primary bone marrow-derived macrophages. Recombinant mouse C3a dose-dependently triggered ERK1/2 phosphorylation in BMDMs (EC_50_ = 8.4 nM) (**Figure 4A**; **Table 1**). Both hTLQP-21 and mTLQP-21 induced ERK signalling in BMDMs (EC_50_ = 96.5 nM for mTLQP-21 and 401 nM for hTLQP-21 respectively), with mTLQP-21 being ~4-fold more potent than hTLQP-21, consistent with the trends observed in CHO-C3aR and HMDMs (**Figure 1B**, **3A**). Neither mTLQP-21 nor hTLQP-21 achieved full activation of C3aR relative to mC3a (63% and 80% for hTLQP-21 and mTLQP-21 respectively). Notably, both TLQP-21 species demonstrated an ~40-fold improvement in potency at murine C3aR (in BMDMs) relative to human C3aR (in HMDMs). The TLQP-21-induced ERK signalling was abolished in C3aR-deficient BMDMs (**Figure 4B**), indicating that the ERK-activating effect was entirely C3aR-dependent. Thus, TLQP-21 similarly activates C3aR-dependent ERK signalling in BMDMs.

## Discussion

TLQP-21 is a neuropeptide with important endocrine and enteroendocrine functions demonstrated by multiple groups (2, 3). Several studies have reported the complement receptor C3aR as an endogenous receptor for TLQP-21 (3, 7, 8, 13, 16). However, few have explored the potential actions of TLQP-21 on immune cells, despite the wide expression of C3aR on these cells (9). Therefore, the primary goal of this study was to characterise the signalling activities of this peptide in primary macrophages, which could allow us to infer whether any of the reported physiological functions of TLQP-21 might be mediated by immune cells in a C3aR-dependent fashion.

First, we validated the signalling activities of our in-house synthesised TLQP-21 on native CHO-K1 cells. In accordance with previous studies (7, 17), both human and mouse TLQP-21 significantly induced ERK1/2 phosphorylation in CHO-K1 cells, with mouse TLQP-21 being marginally more efficacious relative to the human version. Unexpectedly, the ERK signalling activity fell at higher concentrations of TLQP-21, which might reflect the unique characteristic of C3aR-mediated signalling activities in primary cells, as previously observed (18).

On CHO-C3aR cells, which artificially express a high density of human C3aR to enable robust pharmacological characterisation (20), both TLQP-21 peptides behaved as full agonists relative to human C3a. This, together with the lack of activity of TLQP-21 on CHO-C5aR1 cells, provides evidence the ERK signalling activity caused by TLQP-21 was mediated by C3aR rather than the highly conserved C5aR1. TLQP-21 is unlikely to activate other endogenous receptors expressed by CHO cells, either, considering the negligible ERK activity triggered by the ligand. However, this will need to validated in future experiments. The relative potencies of the two versions of TLQP-21 on CHO-C3aR, with the mTLQP-21 being ~ 7-fold more potent than hTLQP-21, are consistent with previous reported data (refer to Figure 5, in Sahu et al. (16)). One possible explanation for the lower potency of hTLQP-21, is that the human peptide has a partially unfolded structure in the presence of hC3aR, whilst mTLQP-21 retains a strong helical structure (16). In addition, by contrast to hTLQP-21, mTLQP-21 possesses a S20A substitution, which confers increased hydrophobicity of the peptide and thereby enhances its binding affinity to the hydrophobic C3aR binding pocket (16, 17). Interestingly, despite the several instances of ligand promiscuity between C3aR and C5a receptors as observed previously (20, 31, 32), we did not observe any significant activity of TLQP-21 on human C5aR2, which adds to the evidence that the action of TLQP-21 was likely to be C3aR-specific.

Previously, TLQP-21 has been shown to induce calcium mobilisation in murine RAW264.7 cells and primary murine microglia (6, 12, 13). However, only mouse TLQP-21 was used in these studies and to the best of our knowledge, no data is available for the potential activity of TLQP-21 in human immune cells. Here, in primary HMDMs, human C3a demonstrated comparable and potent ERK-inducing activities in both HMDMs and CHO-C3aR cells. However, by contrast to hC3a, the agonistic activity of TLQP-21 peptides was markedly weaker in HMDMs relative to CHO-C3aR, with EC_50_ values in the high micromolar range, and neither peptide triggered full activation (agonism), relative to hC3a. Next, we sought to decipher if the induced ERK signalling activity was mediated through C3aR. Because of the impracticality of knocking out C3aR in primary human macrophages, we used SB290157 as a pharmacological tool to block C3aR, which we have demonstrated to be a viable approach in HMDMs (20, 30). SB290157 significantly inhibited C3a-induced ERK signalling, to less than 20% of the untreated level. Considering that SB290157 at the concentration used does not exhibit any detectable agonist activity on C5aR1 or C3aR (20), the observed dampening effect on ERK signalling was likely caused by SB290157 inhibiting C3aR. Next, in our receptor desensitization assay, pre-treating HMDMs with both versions of TLQP-21 was able to dampen C3a-induced ERK1/2 phosphorylation, and expectedly, mTLQP-21 was consistently more potent than hTLQP-21 for all concentrations of hC3a tested. The TLQP-21 mediated inhibitory effect could be overcome by adding higher concentrations of hC3a. Because no such inhibition was observed for C5a-mediated ERK signalling in the same cells, the inhibitory action of TLQP-21 was unlikely to be caused by any non-specific inhibitory actions of the peptide on the cell signalling of HMDMs. Further, the competitive behaviour observed between TLQP-21 and C3a possibly indicate that TLQP-21 and C3a bind to a similar binding site on C3aR, potentially involving D417, R161, and R340 in the C3aR binding motif interacting with the *C*-terminal arginine of TLQP-21 and C3a (16). Together, this provides evidence that TLQP-21 induces ERK1/2 phosphorylation in human macrophages through C3aR.

Human C3aR shares 65% similarity with its murine counterpart. However, the extracellular loop II of human and murine C3aR, which is required for C3a binding, only shares a 45% sequence identity (33). Considering a large number of TLQP-21 studies are performed in mouse models, we examined the signalling activities of TLQP-21 on murine C3aR using murine primary bone marrow-derived macrophages. Consistent with our previous findings in CHO-C3aR and HMDMs and in literature (2, 17), mTLQP-21 more potently induced ERK signalling than hTLQP-21. Neither hC3a, nor the TLQP-21 peptides caused any significant ERK signalling activity in C3aR-deficient cells, confirming the C3aR-specificity of the signalling actions of TLQP-21 in mouse cells.

In this study, by measuring the cell signalling activities of TLQP-21 in native CHO-K1, CHO-C3aR and primary human and murine macrophages, we validated that TLQP-21 also activates and signals through C3aR in a primary immune cell setting. Consistent with previous publications, murine TLQP-21 was more potent than human TLQP-21 on both human and mouse C3aR, and both peptides demonstrated improvement in potency on murine C3aR relative to human C3aR (8, 16). This could be explained by the increased hydrophobicity of the mC3aR binding pocket relative to hC3aR (ILVMS, 59% hydrophobicity for mC3aR versus VSLVC, 53% hydrophobicity for hC3aR), which enables more favourable interaction with *C*-terminal motif of TLQP-21 (PPAR for mTLQP-21 and PPSR for hTLQP-21) (16).

Notably, mTLQP-21 demonstrated a moderate potency in the submicromolar range and close-to-full agonistic potency on mouse BMDMs. By contrast, hTLQP-21 is a weak activator of human C3aR in HMDMs, with a high micromolar potency and only reaching ~50% of the C3a-induced level at the highest dose tested (10 μM). In the physiological context, the plasma concentration of TLQP-21 are reported to be 70 pmol/ml (70 nM) in mouse and 80–90 pmol/ml (80-90 nM) in healthy humans, but much lower in tissues such as the pancreas (34–36). This suggests that in the human system, physiological concentrations of human TLQP-21 acting through C3aR, are unlikely to elicit any significant response (i.e., less than 5% of C3a activity based on our data), at least in peripheral blood macrophages. Indeed, on a molar potency level, hTLQP-21 was ~135,000-fold less active than C3a, which would necessitate a remarkably high local concentration of the parent molecule VGF in order for this peptide to be functional at C3aR *in vivo*. Similar discrepant actions of mTLQP-21 and hTLQP-21 were also reported by Sahu et al. (16), where only mTLQP-21, but not the human counterpart, significantly potentiated 10 nM isoproterenol-induced lipolysis in human adipocytes. The current study is, nevertheless, limited by the primary focus on macrophages. It would be interesting to explore in future experiments whether the weak and partial C3aR activity of hTLQP-21 also holds in other C3aR-expressing cells that may be in close vicinity to VGF-expressing cells within the peripheral and central nervous systems. Further, it is necessary to assess cell signalling pathways other than ERK1/2 phosphorylation, such as cAMP signalling, intracellular calcium mobilisation and β-arrestin recruitment, to gain a more wholistic view of C3aR activity following TLQP-21 ligation. These explorative studies would also help to provide a mechanistic explanation for the partial activity of TLQP-21 on C3aR, and the differential responses between human and mouse C3aR. Alternative signalling pathways in cells other than macrophages (e.g., neutrophils, epithelial cells, neuronal cells), may account for the physiological activity of TLQP-21 reported in the literature.

Importantly, the potential effects of administering TLQP-21 *in vivo* have been explored in a range of mouse models, to explore the biological functions of TLQP-21 in food intake, metabolism, gastric contractility and acid secretion, reproduction, nociception, neuroprotection and modulation of microglia functions (2, 3). Nevertheless, few have examined or validated the biological effects of TLQP-21 in a relevant human system, such as by using human primary cells, tissues or organoids. Considering the large discrepancy in potency and efficacy between hTLQP-21 (on hC3aR) and mTLQP-21 (on mC3aR) in macrophages, further studies are needed to validate if the physiological functions of TLQP-21 observed in mouse models can be translated into human biology. Indeed, one of the major physiological functions of TLQP-21, as reported by several groups, is to modulate microglia functions, which subsequently has implications in β-Amyloid plaque clearance, Alzheimer’s disease neuropathy and nociception (6, 11, 12, 37). As human primary microglia cell models and 3D cultures become more widely available (38–40), it would be informative to examine any modulatory effect of TLQP-21 in these human systems.

In sum, this study characterised the signalling activities of TLQP-21 on human and murine primary macrophages and demonstrated the ability of both human and mouse TLQP-21 to activate C3aR-dependent ERK signalling, with the mouse TLQP-21 being consistently more potent than the human counterpart. TLQP-21 confers important physiological functions. However, considering the supraphysiological concentrations of hTLQP-21 needed to only partially activate human macrophages, it is conceivable that any immune actions of TLQP-21 are mediated by pathways other than C3aR in humans.

## Data availability statement

The raw data supporting the conclusions of this article will be made available by the authors, without undue reservation.

## Ethics statement

The studies involving human participants were reviewed and approved by The University of Queensland Human Research Ethics Committee. The patients/participants provided their written informed consent to participate in this study. The animal study was reviewed and approved by The University of Queensland Animal Ethics Committee.

## Author contributions

TW and XL conceived the project and designed the research. XL performed the research, analysed data and wrote the first paper draft with assistance from TW. JL generated the murine cells used in this study. HL and RC synthesised the TLQP-21 peptides and other key reagents for the study. All authors contributed to the article and approved the submitted version.
